# Considering Litter Effects in Preclinical Research: Evidence from E17.5 Acid-Sensing Ion Channel 2a Knockout Mice Exposed to Acute Seizures

**DOI:** 10.3390/brainsci15080802

**Published:** 2025-07-28

**Authors:** Junie P. Warrington, Tyranny Pryor, Maria Jones-Muhammad, Qingmei Shao

**Affiliations:** 1 Department of Neurology, University of Mississippi Medical Center, Jackson, MS 39216, USA; 2 Program in Neuroscience, University of Mississippi Medical Center, Jackson, MS 39216, USA

**Keywords:** litter effect, ASIC2a, seizure, uterine position, sex differences

## Abstract

**Background:** The reproducibility of research findings continues to be a challenge in many fields, including neurosciences. It is now required that biological variables such as sex and age be considered in preclinical and clinical research. Rodents are frequently used to model clinical conditions; however, litter information is rarely presented. Some studies utilize entire litters with each animal treated as an independent sample, while others equally assign animals from each litter to different groups/treatments, and others use averaged data. These methods can yield different results. **Methods:** This study used different analysis methods to evaluate embryo and placenta weights from E17.5 acid-sensing ion channel 2a (ASIC2a) mice with or without seizure exposure. **Results:** When each embryo was treated as an individual sample, fetal and placental weight significantly differed following seizures in the ASIC2a heterozygous (+/−) and homozygous (−/−) groups. Differences in fetal weight were driven by females in the ASIC2a+/− group and both sexes in the ASIC2a−/− group. These differences were lost when an average per sex/genotype/litter was used. There was no difference in placental weight when treated individually; however, female ASIC2a−/− placentas weighed less following seizures. This difference was lost with averaged data. ASIC2a−/− fetuses from −/− dams had reduced weights post-seizure exposure. Position on the uterine horn influenced embryo and placental weight. **Conclusions:** Our results indicate that using full litters analyzed as individual data points should be avoided, as it can lead to Type I errors. Furthermore, studies should account for litter effects and be transparent in their methods and results.

## 1. Introduction

Rodents are the most widely used animal models in preclinical studies, partly because they are multiparous and can produce multiple offspring with each pregnancy, allowing for many animals to be generated and quick turnaround of studies. This advantage of multiparous pregnancies can add to the complexity of study design that promotes rigor and reproducibility. Funding agencies such as the National Institutes of Health and scientific journals have now required sex to be considered as a biological variable. There is strong evidence that sex is an important biological variable that should be considered in all studies [1], and there are publications addressing how to implement sex as a biological variable in research [2,3]. However, other variables such as litter effects are less widely addressed and are not currently required biological variables to be considered and reported in manuscripts and grants. Some fields, such as neurodevelopment, have addressed the importance of considering litter effects in neurodevelopmental studies [4,5,6,7], but not much has been done in cardiovascular and general neuroscience research studies. Moreover, while it is generally discussed in the pregnancy field, empirical primary data addressing the litter effects are lacking. Thus, in this study, we assessed litter as a biological variable.

For the current study, we utilized a knockout model and seizure exposure as additional factors to show how manipulating litter-specific parameters would impact results. Thus, the ASIC manipulation and seizures are tools rather than the focus of the current study. Acid-sensing ion channels (ASICs) are part of the Degenerin family of proteins, function as chemo- and mechano-sensors, and are activated by drops in extracellular pH [4,5,6]. In a previous study by our group, we showed that reduced ASIC2a leads to worse drug-induced seizures in pregnant females compared to wild-type pregnant controls [7]. The effect of this acute seizure exposure, genotype, or fetal sex on the developing embryo and placenta was not addressed. To our knowledge, there are no published reports exploring this.

Studies have linked maternal seizures to intrauterine growth restriction, low birth weight, and premature birth in humans [8,9], although these studies were based on the chronic seizure disorder of epilepsy. The current study utilized a very acute exposure of 30 min, mimicking what might happen in the context of eclampsia, an acute pregnancy disorder [10] with novel manifestations of seizures during pregnancy. Furthermore, we chose to induce seizures using pentylenetetrazol because it is one of the most common pro-convulsant drugs used in pregnancy studies [7,11,12,13,14,15,16].

In addition to sex, genetic manipulation, number of fetuses, and maternal exposure, position on the uterine horn can also influence fetal characteristics [17], although findings are mixed. Specifically, one study performed in pigs showed that fetuses near the cervical end were lighter and those closest to the ovaries were heavier [17]. Another study showed that depending on the location of the fetus on the uterine horn, there was a significant difference in fetal weight in those exposed to titanium dioxide nanoparticles, with differences found in those located at the center of the uterine horn [18]. The position of pups along the uterus influences fetal weight and length, with pups closest to the ovaries and cervix being heavier than those in the center.

In the current study, we used a systematic approach to explore whether litter effects contribute to changes in embryo and placenta weight in mice with genetic manipulation of ASIC2a and following exposure to acute seizures experienced by the dam. We treated each placental-fetal unit as an independent unit in one analysis, and then performed the same comparisons when the litter was the experimental unit (averaged data per litter). We also assessed the contribution of maternal genotype and uterine position on embryo and placenta weight in mice with wild-type, heterozygous, or homozygous (knockout) expression of ASIC2a. Our study is unique because it includes multiple different analyses to systematically probe the effect of litter variables on fetal and placental weight while accounting for acute exposure to seizures. To our knowledge, our study is the first to employ this level of detailed analysis of litter effects in utero.

## 2. Materials and Methods

### 2.1. Animals

Heterozygous B6.129-Asic2tm1Wsh/J mice (Jackson Laboratory, Bar Harbor, ME, USA, JAX Mice stock number: 013126, RRID: IMSR_JAX:013126) [19] were bred in the Lab Animal Facilities at the University of Mississippi Medical Center (UMMC) to generate genotypes for the experiments. Dams were fed a standard rodent diet (Teklad 22/5 Rodent Diet 8640) and water ad libitum until breeding, when they were switched to a breeders’ diet (Teklad Global 19% Protein Extruded Rodent Diet 2019) from mating until euthanasia at gestational day (GD) 18.5. Mice were housed 2–5 per cage at a constant temperature of ~74 °F and on a 12 h light and 12 h dark cycle. Pregnant mice (aged 3–5 months) had access to paper huts as environmental enrichment. All procedures were approved by the UMMC Institutional Animal Care and Use Committee (Protocol: 1434A, approved 6 December 2017, and 1434B, approved 30 November 2020) in accordance with the 8th edition of the National Research Council Guide for Care and Use of Laboratory Animals.

### 2.2. ASIC2a Genotyping

ASIC2a genotyping was performed using ear punches of dams and breeder males at weaning. The ASIC2a genotype and genetic sex of each embryo were determined using tail snips at the end of the experimental protocol (E17.5). DNA extraction and PCR genotyping were performed using the KAPA Mouse Genotyping Kit (KAPABiosystems, Cat#:KR0385_S-v2.17). The tail snips were added to the KAPA Expression Extraction Buffer, and lysed at 75 °C for 10 min, followed by enzyme inactivation at 95 °C for 5 min. The lysate was centrifuged for 5 min, and the supernatant was stored for genotyping. The following sequences were used for ASIC2a genotyping (5′-3′): Reaction A: AGT CCT GCA CGG TGG GAG CTT CTA; GAA GAG GAA GGG AGC CAT GAT GAG, Reaction B: ATG GTT TCG GAG TGG TTT GGC ATT GTG; TGG ATG TGG AAT GTG TGC GA. PCR using Zfy primers confirmed the presence of the Y chromosome, using the following primers for Zfy-F: 5′GAC T A GACATGTCTTAACATCTGTCC-3′ and Zfy-R: 5′-CC T ATTGCATGGACTGCAGCTTATG-3′ [20]. PCR products (5 µL), GelRed Nucleic Acid Stain (Biotium Cat#: 41003, 3.5 µL), and 6 µL of 100 base DNA ladder (Fisher Cat#: BP2573-100), were loaded on 2% agarose gels and electrophoresed at 140 V for 45 min. Gels were imaged using the ChemiDoc MP system (Bio-Rad). The heterozygous mice produce bands that migrate to 365 and 450 bp, with the wild-type band at 365 bp and the mutant band at 450 bp. For genetic sex, males were identified by the presence of a 198 bp band, while females did not exhibit this band.

### 2.3. Breeding and Establishment of Timed Pregnant ASIC2a Mice

One to 3 female mice were paired with one male of the same age and genotype in the evening. The following day, male mice were returned to their home cages. As mice were paired for only 1 night, the exact date of pregnancy is known. The day of separation was considered as GD 0.5. Mice were monitored for changes in abdominal size, and pregnancy was confirmed on GD 13.5. Mice that did not become pregnant were subjected to one additional round of mating. All pregnant mice were included in the study.

### 2.4. Seizure Induction

On GD 17.5, pregnant ASIC2a wild-type (+/+) (n = 7), heterozygous (+/−) (n = 14), and homozygous (−/−) (n = 6) mice were administered 40 mg/kg of pentylenetetrazol (PTZ) via intraperitoneal (i.p.) injection. No a priori power analysis was performed. After injection, mice were video-monitored for 30 min. Pregnant mice not subjected to seizures were injected with Evans blue dye, which was left to circulate for 30 min. Mice were euthanized immediately after observation or circulation under isoflurane anesthesia (SomnoSuite, Kent Scientific). Because behavioral seizures were being assessed in the dams, no interventions were performed to reduce pain and distress during the seizure recording. Investigators conducting the seizure induction and behavior analysis in the dams were unaware of the maternal genotype. Furthermore, the sex and genotype of offspring were unknown until after genotyping (days after tissue collection). All resulting offspring were included in the study and used in the analyses.

### 2.5. Euthanasia and Tissue Processing

Under isoflurane anesthesia, maternal blood was collected via cardiac puncture, and the heart was removed. The number of live and resorbed embryos was counted, and live embryos and placentas were weighed. The position of each embryo and placenta on the uterine horn was noted for later identification.

### 2.6. Statistics and Data Analysis

All embryos from the pregnant dams were included in the analysis, and none were excluded. We analyzed the resulting embryo and placenta weight data using different methods:

1. Individual embryo/placenta treated as an independent sample. In this analysis model, individual embryos and placentas were grouped based on genotype, sex, and/or seizure exposure and analyzed.
2. Averaged data per litter: To calculate embryo and placental weights, measurements were averaged to obtain mean data per sex per genotype for each litter. These data were then reported per dam in each figure.
3. Averaged data per litter grouped by maternal genotype. Averaged data were further grouped based on maternal genotype, resulting in 5 groups: ASIC2a+/+ embryos from ASIC2a+/+ dams, ASIC2a+/+ embryos from ASIC2a+/− dams, ASIC2a+/− embryos from ASIC2a+/− dams, ASIC2a−/− embryos from ASIC2a−/− dams, or ASIC2a−/− embryos from ASIC2a+/− dams.
4. Individual embryo/placenta as independent samples grouped by genotype and position on the uterine horn.

A three-way analysis of variance was used to assess the main effects of sex, genotype, and seizure exposure on the endpoints. Two-way ANOVA or Mixed Effects ANOVA was used to analyze the main effects of genotype and seizure exposure, followed by Sidak post hoc analysis. All analyses and graphs were performed or generated using GraphPad Prism (v. 10.5.0). Comparisons with *p* < 0.05 were considered statistically significant.

## 3. Results

### 3.1. Effect of ASIC2a Genotype and Acute Seizure Exposure on Embryo Weight

**Figure 1:**
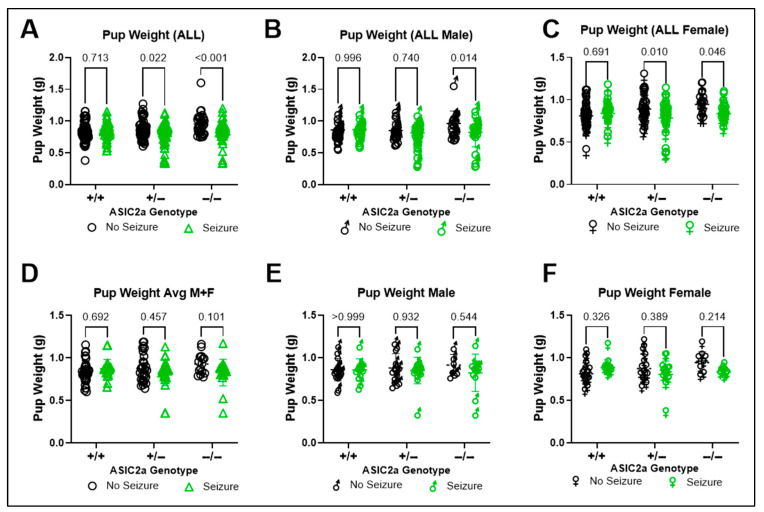
**Changes in embryo weight at E17.5.** Pup weight was (**A**) significantly lower in ASIC2a+/− and −/− mice exposed to seizures when individual embryos were plotted regardless of litter; (**B**) lower in −/− males following seizure exposure; (**C**) lower in +/− and −/− females following seizure exposure (n = 34–96). Average embryo weight was unchanged following seizure exposure in (**D**) combined sex, (**E**) male, or (**F**) female embryos (n = 16–36). Dots represent individual embryos (**A**–**C**) or average embryo weight per litter (**D**,**E**). *p*-values are indicated above respective pairwise comparisons. Data analyzed using Two-Way ANOVA followed by Sidak’s Multiple Comparison test.

We first assessed the impact of seizure and ASIC2a genotype on fetal weight at E17.5 using all offspring from each litter. There was a significant interaction between seizure exposure and genotype on fetal body weight (F(2, 391) = 7.615; *p* < 0.001) and a significant effect of genotype (F(2, 391) = 3.947; *p* = 0.020) and seizure exposure (F(1, 391) = 13.06; *p* < 0.001) on fetal body weight. Specifically, significant differences were found in the ASIC2a+/− (*p* = 0.022) and ASIC2a−/− (*p* < 0.001) offspring with a reduction in body weight following acute seizure exposure (Figure 1A). Because some studies select one sex for further studies, we separated the body weights based on sex. In males, a significant interaction (F(2, 192) = 3.051; *p* = 0.050) and effect of seizure exposure was obtained (F(1, 192) = 5.623; *p* = 0.019) with significant differences in the −/− male offspring (Figure 1B, *p* = 0.014). In females, there was a significant interaction between seizure exposure and genotype (F(2, 193) = 5.497; *p* = 0.005) and a trend for a main effect of genotype (F(2, 193) = 2.940; *p* = 0.055) and seizure exposure (F(1, 193) = 8.070; *p* = 0.005) on body weight. In females, reduced body weight was observed in +/− (*p* = 0.010) and −/− (*p* = 0.046) fetuses (Figure 1C). We then analyzed the average body weight per sex per litter to determine whether litter effects influenced the observed differences. There was no significant interaction between or main effects of genotype or seizure exposure on fetal body weight (Figure 1D). Moreover, when the averaged data were separated based on sex, there was no difference in males (Figure 1E) or females (Figure 1F). However, a significant interaction between genotype and seizure exposure (F(2, 72) = 3.542; *p* = 0.034) was found.

### 3.2. Effect of ASIC2a Genotype and Acute Seizure Exposure on Placenta Weight

**Figure 2:**
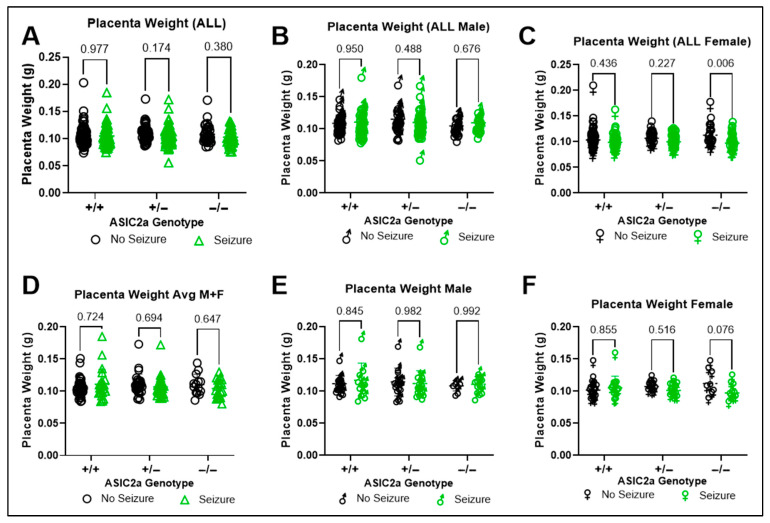
**Changes in placenta weight at E17.5.** Placenta weight was (**A**) not different within genotypes after seizure exposure when individual pups were plotted regardless of litter; (**B**) not different in males following seizure exposure; (**C**) lower in ASIC2a−/− females following seizure exposure (n = 34–96). Average placenta weight was unchanged following seizure exposure in (**D**) combined sex, (**E**) male, or (**F**) female placentas (n = 16–36). Dots represent individual pups (**A**–**C**) or average placenta weight per litter (**D**,**E**). *p*-values are indicated above respective pairwise comparisons. Data analyzed using Two-Way ANOVA followed by Sidak’s Multiple Comparison test.

We then looked at the effect of genotype and seizure exposure on placental weight. There was no significant interaction (F(2, 391) = 0.852; *p* = 0.427) or main effect of genotype (F(2, 391) = 1.033; *p* = 0.357) on placental weight. There was a main effect of seizure exposure on placenta weight (F(1, 391) = 4.803, *p* = 0.029) when all placentas from each litter were included in the analysis (Figure 2A). In male placentas, there was no effect of genotype (F(2, 192) = 1.491; *p* = 0.228) or seizure exposure (F(1, 192) = 0.056; *p* = 0.813) on placental weight (Figure 2B). In female placentas, seizure exposure significantly reduced placental weight (F(1, 193) = 14.08; *p* < 0.001) with a significant reduction in −/− placentas (Figure 2C, *p* = 0.006). Next, we assessed the same effects using averaged data. There was no effect of genotype or seizure exposure on placental weight (*p* > 0.05) when males and females were combined (Figure 2D). Moreover, there was no effect of genotype or seizure exposure on male or female placental weight (Figure 2E,F).

### 3.3. Influence of Maternal Genotype on Embryo and Placenta Weight

**Figure 3:**
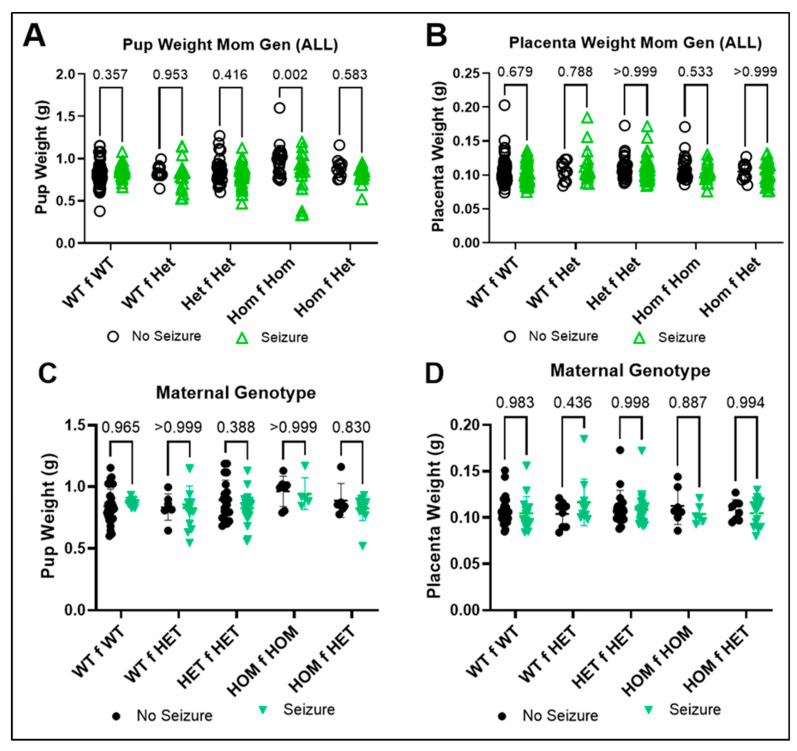
**Effect of maternal genotype on embryo and placenta weight at E17.5.** Individual embryo (**A**) and placenta (**B**) weights are shown based on maternal genotype (n = 11–86). Averaged data per genotype per litter for (**C**) embryo and (**D**) placental weight (n = 5–29). Dots represent individual embryos (**A**,**B**) or average placenta weight per litter (**C**,**D**). *p*-values are indicated above respective pairwise comparisons. Data analyzed using Two-Way ANOVA followed by Sidak’s Multiple Comparison test. WT = wild-type, HET = heterozygous, and HOM = homozygous. The first genotype represents the pup genotype, while the second genotype represents the dam genotype. For example, WT f WT represents wild-type pup from wild-type dam.

The embryo and placenta weights were grouped based on maternal genotype to determine whether maternal genotype influenced embryo and placenta weight. There was a significant interaction between genotype and seizure exposure (F(4, 334) = 4.265; *p* = 0.001), a significant effect of seizure exposure (F(1, 334) = 8.404; *p* = 0.004), and a trend for significant effect of genotype (F(4, 334) = 2.187; *p* = 0.070) on embryo weight. Homozygous knockout embryos from −/− dams had a significantly lower body weight following seizure exposure than non-exposed embryos (Figure 3A, *p* = 0.002). There was no significant effect of genotype or seizure-exposure on placental weight when grouped by maternal genotype (Figure 3B, *p* > 0.05). The mean embryo weight per sex per genotype was grouped based on maternal genotype. There was no effect of genotype or seizure exposure on embryo weight (Figure 3C, *p* > 0.05). Thus, whether the +/+ embryo arose from a +/+ or +/− dam, there was no significant difference in embryo weight. A similar finding was observed for placenta weight (Figure 3D, *p* > 0.05).

### 3.4. Influence of Embryo and Placenta Location on the Uterine Horn on Embryo and Placenta Weight

**Figure 4:**
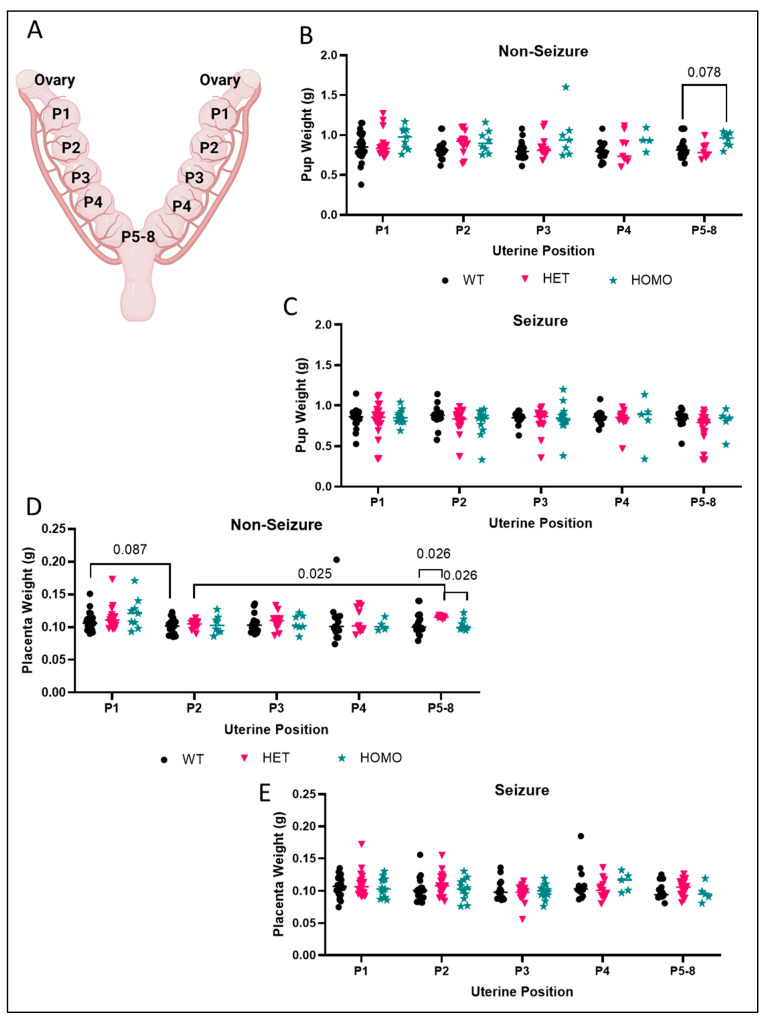
**Position on the uterine horn has a modest effect on embryo weight and no influence on placenta weight.** (**A**) Schematic of the uterine horn used to define embryo location. Left and right uterine horns were not separated from analysis due to small sample sizes for some positions. The schematic of the position of the uterine horn was created using BioRender.com. (**B**) In embryos not exposed to seizures, homozygous pups had increased weight compared to wild-type (n = 2–27). (**C**) No difference in pup weight between genotypes and within uterine positions following seizure exposure (n = 5–21). Dots represent individual embryo weight (**A**–**C**) or individual placenta weight (**D**,**E**). *p*-values are indicated above respective pairwise comparisons. Data analyzed using Two-Way ANOVA followed by Sidak’s Multiple Comparison test.

A schematic for defining embryo location is shown in Figure 4A. There was a main effect of genotype on embryo weight (Figure 4B) in the non-seizure exposed pups when grouped based on position on the uterine horn (F(2, 48) = 6.487; *p* = 0.003). At uterine position P5-8, there was a trend for an increase in body weight of −/− embryos compared to +/+ embryos (*p* = 0.078) and −/− compared to +/− (*p* = 0.057). No significant effects of uterine position or genotype were obtained for embryo weight in the seizure-exposed groups (Figure 4C). We then looked at the effect of uterine position on placenta weight. We show that uterine position had a main effect on placenta weight (F(2.933, 93.87) = 2.743; *p* = 0.049) with a trend for decreased placenta weight in +/+ placentas between positions 1 and 2 on the uterine horn (*p* = 0.087). A significant increase in placenta weight was observed in +/− offspring at positions 5–8 vs. P2 on the uterine horn (*p* = 0.025; Figure 4D). Additionally, at position 5–8 (closest to the cervix), +/− placentas were heavier than +/+ (*p* = 0.026) and −/− (*p* = 0.026) placentas. In seizure-exposed placentas, a significant main effect of uterine position was found (F(3.420, 124.0) = 2.818; *p* = 0.035) with no significant pairwise differences between genotypes or positions (*p* > 0.05; Figure 4E).

### 3.5. Effect of Seizure Exposure on Embryo Weight Within Uterine Positions

**Figure 5:**
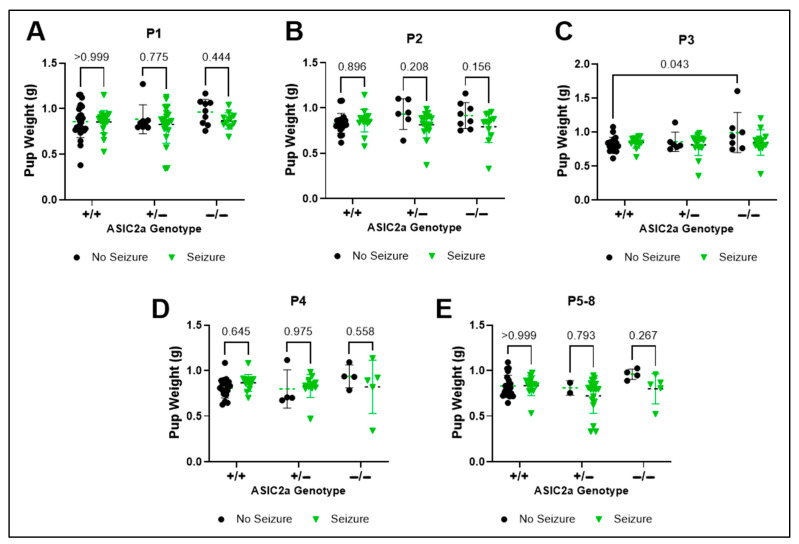
**No difference in embryo weight following seizure exposure.** Pup weight was not different at (**A**) P1, (**B**) P2, (**C**) P3, (**D**) P4, or (**E**) P5-8 between non-seizure exposed and seizure exposed pups (n = 2–27). Dots represent individual pup weight. *p*-values are indicated above respective pairwise comparisons. Data analyzed using Two-Way ANOVA followed by Sidak’s Multiple Comparison test.

To assess the effect of genotype and seizure exposure on embryo weight at each uterine horn location, we directly compared seizure vs. non-seizure exposed pups at each uterine position. There was no effect of genotype F(2, 99) = 1.133; *p* = 0.325 or seizure F(1, 99) = 2.685; *p* = 0.105) on embryo weight at P1 (Figure 5A). There was a trend for an interaction between genotype and seizure exposure (F(2, 80) = 2.822; *p* = *0*.065) and a trend for a seizure effect at P2 (F(2, 80) = 3.206; *p* = 0.077; Figure 5B). At position 3, non-seizure-exposed −/− embryos weighed more than +/+ embryos (*p* = 0.046; Figure 5C). There were no effects of seizure or genotype on fetal weight at P4 (Figure 5D; *p* > 0.05), but a significant effect of seizures on embryo weight at P5-8 (F(1, 67) = 4.261; *p* = 0.043; Figure 5E).

### 3.6. Effect of Seizure Exposure on Placenta Weight Within Uterine Positions

**Figure 6:**
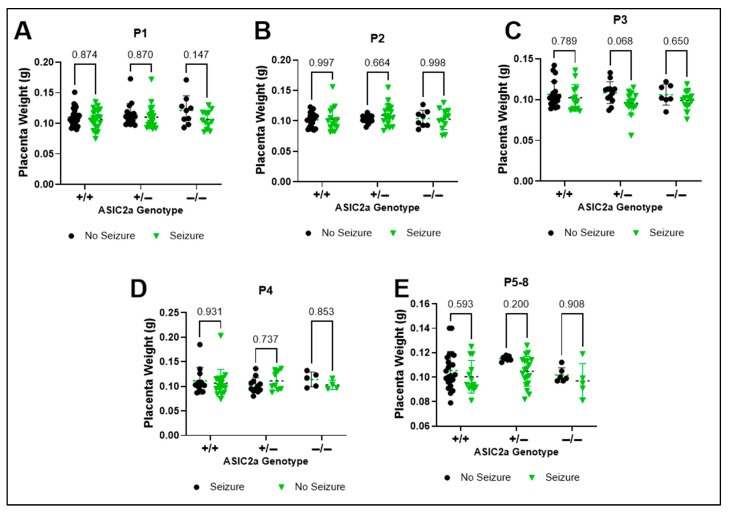
**No difference in placenta weight following seizure exposure.** Placenta weight was not different at (**A**) P1, (**B**) P2, (**C**) P3, (**D**) P4, or (**E**) P5-8 between non-seizure exposed and seizure exposed placentas (n = 2–27). Dots represent individual placenta weights. *p*-values are indicated above respective pairwise comparisons. Data analyzed using Two-Way ANOVA followed by Sidak’s Multiple Comparison test.

To assess the effect of genotype and seizure exposure on placenta weight at each uterine horn location, we directly compared seizure vs. non-seizure exposed placentas at each uterine position. There was a trend for a significant effect of seizure exposure at P1 on placenta weight F(1, 97) = 3.558; *p* = 0.062 and no genotype effect F(2, 97) = 1.212; *p* = 0.302) on placenta weight at P1 (Figure 6A). There was no seizure (F(1, 80) = 0.348; *p* = 0.557) or genotype (F(2, 80) = 0.655; *p* = 0.522) effect at P2; Figure 6B) At P3, there was a significant seizure effect (F(1, 72) = 5.356; *p* = 0.024; Figure 6C) and no effects of seizure or genotype on placental weight at P4 (Figure 6D). At P5-8, there was a significant effect of seizure exposure (F(1, 67) = 4.401; *p* = 0.040) and a trend for a genotype effect (F(2, 67) = 2.874; *p* = 0.063) effect on placenta weight (Figure 6E).

### 3.7. Effect of Litter Size on Fetal Weight

**Figure 7:**
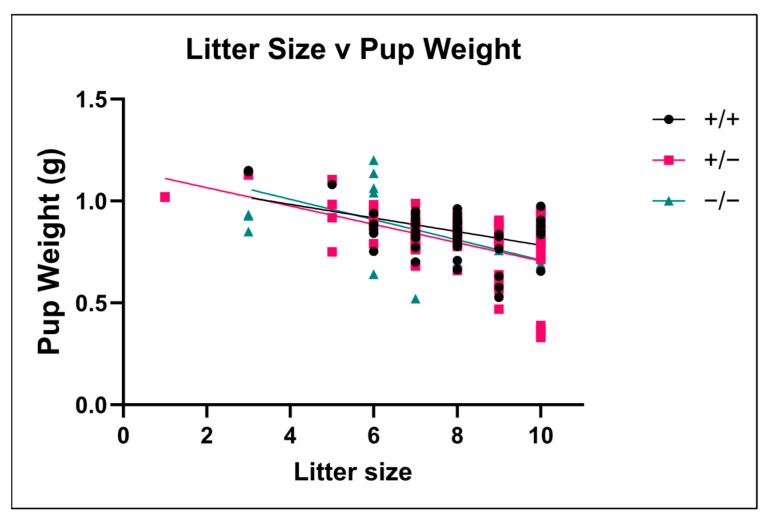
**The relationship between litter size and embryo weight.** Embryo weights were plotted against litter size for each genotype. Larger litters had the lowest embryo weights. Each point represents an embryo. Data were analyzed using linear regression.

To assess the relationship between litter size and embryo weight, we plotted the embryo weights from each litter against the total litter size (Figure 7). There was a significant negative correlation between embryo weight and litter size for all three genotypes. In ASIC2a +/+ (r^2^ = 0.193, *p* < 0.001), +/− (r^2^ = 0.251, *p* < 0.001), and −/− (r^2^ = 0.247, *p* < 0.001) embryos, smaller litters had the heaviest embryos.

## 4. Discussion

In embryonic studies, it is challenging to differentiate between male and female offspring via visual observation, although not impossible. Investigators are left with choices that include: (1) randomly selecting embryos without knowing the sex or genotype, (2) utilizing all embryos present and including them in the study as individual data points, (3) genetically identifying sex and genotype post-collection and averaging the data so that each data point represents averaged litter data, or (4) selecting 1 of each sex per genotype randomly after genetic determination of sex and genotype. In the current study, we compared 2 of the analysis options to determine whether different analysis methods resulted in different findings. We report that litter effects could influence basic characteristics such as embryo and placenta weight during late embryonic stages (E17.5). When embryos were treated as individual data points, a greater statistical power was achieved, leading to significant interactions, main effects, and pairwise differences in both embryo and placenta weight. These differences were not maintained when average data were used.

Embryo, placenta, and birth weight are good predictors of future cardiovascular health. One of the seminal studies linking birth weight to later cardiovascular risk was performed by Barker and colleagues [21] using a male-only cohort. The same group went on to show a strong link between adult hypertension and birthweight, head circumference, and other placental characteristics in a cohort of middle-aged males and females [22]. Together, these studies demonstrated that birth/gestational parameters can influence long-term cardiovascular health.

The current study shows that treating offspring within a litter as independent data points can skew the data and increase the likelihood of finding a statistically significant difference. There is evidence from the literature that the size of the litter is inversely associated with fetal weight [23]. Our current analysis supports these findings, with smaller litters having higher overall body weight regardless of genotype. Because we utilize embryos, our study cannot assess whether the differences observed in utero will continue into adulthood and whether litter parameters influence adult cardiovascular parameters. Evidence supporting the hypothesis that fetal or birth weight can influence future cardiovascular health has been provided through studies linking pregnancy complications to long-term cardiovascular risks.

Intra-uterine growth restriction (IUGR) and small for gestational age fetuses are common features of hypertensive disorders of pregnancy, such as preeclampsia. Using rodent models of preeclampsia, studies have demonstrated that IUGR leads to hypertension in adulthood [24,25,26]. Furthermore, IUGR is associated with neurocognitive impairment in childhood [27,28] and late adulthood [29,30]. Therefore, birthweight is an important biological factor that should be carefully controlled via eliminating litter effects or selecting specific weight targets when assigning to groups/treatments. These details should then be outlined in any publication’s study design/experimental approach section.

Studies assessing the impact of maternal seizures during pregnancy on birthweight are complex. Studies have assessed birthweight in women with epilepsy and have found increased risk of low birth weight and preterm delivery, and a further increased risk in epilepsy patients who experienced seizure during pregnancy [31]. Another study showed that women with epilepsy are more likely to have a low birthweight offspring, and that IUGR was associated with anti-epilepsy drug treatment [32,33]. While these studies indicate reduced birthweight in women with epilepsy, the condition of epilepsy is chronic, unlike our study, where the pregnant dam experienced a single, acute seizure exposure. Our study shows that even acute seizure exposure reduces embryo and placenta weight in exposed offspring at E17.5. We utilized pentylenetetrazol as the seizure-inducing agent because it is commonly used in pregnancy studies [7,11,12,13,14,15,16]. It is possible that the use of other agents, such as kainic acid, lithium, and pilocarpine, may yield different results.

Because the maternal uterine environment influences fetal growth and health, we assessed whether the maternal genotype influenced embryo and placenta weight and the response to seizure exposure. We found that ASIC2a−/− embryos from −/− dams had lower body weight after seizure exposure when all embryo data were used. However, the differences were no longer significant when the averaged data were utilized. These data demonstrate that homozygous embryos from homozygous dams are more sensitive to seizure exposure than those from heterozygous dams. Therefore, researchers must consider the genotype of the dams generating the offspring to be used in various studies.

Another factor sometimes ignored in offspring data is position on the uterine horn. Studies assessing the relationship between uterine position and embryonic weight are reported more frequently in pigs. One study showed that heavier fetuses were more likely to be located close to the tubal ends (P1-2 in the current study, Figure 4A) and the lighter fetuses closer to the cervix (P5-8 in the current study) [17]. We report that the heaviest placentas were located near the cervix (P5-8) of non-seizure exposed pregnancies when all genotypes were combined, which is different from the results from pigs.

The current study has several strengths. First, we considered the effect of multiple factors/variables on two fundamental biometric factors with strong association to long-term cardiovascular and other risks—embryo and placenta weight. We considered litter effects, maternal genotype, in utero position on the uterine horn, and litter size. We also considered how maternal experience of adverse events, such as seizures, can influence biometrics of the placental-fetal unit even when the experience/exposure was acute. To our knowledge, this is one of the first studies to perform a rigorous analysis such as this.

**Figure 8:**
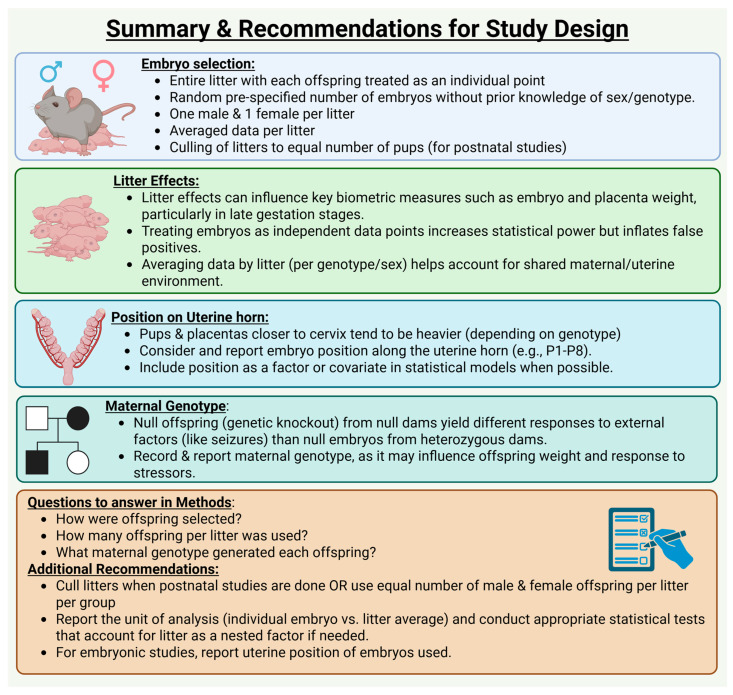
Summary of findings and recommendations for studies involving animals.

There are some limitations with our study design. First, while we detected differences in embryo and placental weight in utero, whether these differences are associated with cardiovascular consequences and long-term physiological abnormalities was not investigated. In the current study, we did not explore the mechanisms for the seizure-exposure induced differences in embryo and placenta weights. Future studies will address how acute seizures in the dam influence changes in embryo and placenta weights in utero. Another potential limitation of the current study is that the sample sizes for the dams used to generate embryos were not consistent, with some groups having smaller sizes, leading to differences in the number of embryos for each genotype generated. Despite this, we were able to detect significant differences. It will also be interesting to see if the litter effects observed in the current study will be replicated in other strains or species. It should also be noted that multi-fetal pregnancies are less common in humans, so to mimic human conditions better, consideration of litter effects is imperative (Figure 8).

## 5. Conclusions

In conclusion, litters should be treated as a single unit rather than broken down into independent individual fetal–placental units. In in utero analyses, investigators should consider location on the uterine horn as a variable and should select from similar locations or should randomize as best as possible. When random selection cannot be performed and several offspring per litter are assigned to groups/treatments, average data per sex and genotype per litter should be used. This will eliminate the litter effect. Additionally, researchers using rodent models should consider litter size and cull litters to similar sizes when applicable. Moreover, while the position on the uterine horn cannot be determined after delivery, researchers should consider uterine position as a potential source of variability and outline the selection method used when assigning rodents to experimental groups. Importantly, when ordering rodents from commercial sources, researchers should inquire about and note whether the animals received were from mixed or single litters, and ensure equal assignment of rodents from each litter to each group/treatment. Considering all these variables and reporting them in the methods section of studies will increase the chances of reproducing reported research findings.

## Data Availability

The original contributions presented in this study are included in the article. Further inquiries can be directed to the corresponding author.

## Abbreviations

The following abbreviations are used in this manuscript:

ASIC2a	Acid-sensing ion channels
GD	Gestational day
PTZ	Pentylenetetrazol
IUGR	Intrauterine Growth Restriction
